# Redirection of transfusion waste and by-products for xeno-free research applications

**Published:** 2019-11-09

**Authors:** Vanessa Zammit, Mark Farrugia, Byron Baron

**Affiliations:** ^1^National Blood Transfusion Service, St. Luke’s Hospital, G’Mangia, PTA1010, Malta; ^2^Centre for Molecular Medicine and Biobanking, Faculty of Medicine and Surgery, University of Malta, Msida, MSD2080, Malta

**Keywords:** fibrin clots, human plasma, platelet lysate, stem cell research, transfusion products, white blood cell cultures, xeno-free culturing

## Abstract

**Background and Aim:**

Whole blood is processed to derive a red cell concentrate, plasma, and buffy coat (BC) (from which platelets can be further extracted). Unused plasma and BCs are common in most blood establishments and considered a liability. The redirection of these products to xeno-free applications is not complicated or time-consuming and cannot only benefit the research recipients but also the blood establishment suppliers interested in research collaboration. The aim of this study is to describe a diverse yet by no means an exhaustive list of options for preparing blood products for research applications.

**Materials and Methods:**

Plasma and BCs from healthy donors were processed using basic laboratory techniques under aseptic conditions and tested for their ability to support the culture of mesenchymal stem cells in both 2D and 3D cultures using fibrin clots. The white blood cells (WBC) from the BCs were induced by phytohemagglutinin and CD marker expression was monitored using quantitative polymerase chain reaction.

**Results:**

All the methods tested for preparing blood products were successful but the applicability to different settings varied greatly with the most successful being the supplementation of Dulbecco’s Modified Eagle Medium: Nutrient Mixture F-12 with 20% cryodepleted plasma and 0.1 mg/mL platelet lysate, the formation of fibrin clots using a ratio of 3:1 (medium: plasma) and the culturing of WBCs with 5 µg/mL phytohemagglutinin.

**Conclusions:**

Using the wastes and by-products of blood establishments for xeno-free cell culturing of stem cells will reduce the reliance on commercially available, ready-made products, and increasing the potential for therapeutic stem cell research. Despite the benefits presented both in terms of cost and applications, further characterization and optimization of each blood product for reproducibility of results is required.

**Relevance for Patients:**

The availability of low-cost xeno-free reagents will speed up therapeutic stem cell research and allow patients to receive treatments of the expected high standards at lower costs.

## 1. Introduction

Over the years, blood transfusion has evolved into one of the most common and simple medical procedures. The concept of processing whole blood to derive blood components has further provided the opportunity to provide patients with products that are fit for use, i.e., each product is individually transfused according to the patient’s requirements. Once processed, whole blood is divided into three components: Red cell concentrate (RCCs), fresh frozen plasma (FFP), and buffy coat (BC). It is possible to produce an additional product, platelets, by further processing of the BC component [1]. Although transfusions are in high demand, it is normal for a blood establishment to have blood components, such as plasma and BCs, that are not transfused or used. If not used, these products are normally discarded, making these a liability for the establishment. A way of preventing this wastage is to redirect the use of these products toward research. This argument can be applied to both plasma and platelets, which have a respective shelf-life of 1.5-3 years (depending on the storage temperature) and 5 days, after which these can no longer be transfused [2]. A cellular blood by-product that is considered waste and never used for transfusion is white blood cells (WBCs) which are entrapped by a special filter to produce leuko-depleted transfusable blood components.

All research with the scope of therapeutic applications should use xeno-free products because it is well-known that animal-derived sera are not well-defined and induce early stage cell differentiation [3]. Once redirected for research, particularly for stem cell culture, plasma can be used both as a replacement for animal-derived sera supplements and for 3-D cell culture. Platelets can be lysed to supplement medium with multiple growth factors such as platelet-derived growth factor, transforming growth factor (TGF), platelet factor interleukin, platelet-derived angiogenesis factor, vascular endothelial growth factor, epidermal growth factor, insulin-like growth factor, and fibronectin [4]. While BCs have long been used as a source for cell isolation for research purposes, this is only limited to institutions having specific collaborations with a blood establishment. Moreover, BCs are mainly used as a source of peripheral blood mononuclear cells for further isolation of cells of interest, and the fact that BCs are a good source of all leukocytes is generally ignored. The use of BC-derived leukocytes can be employed as models in the research of the immune system to study immune interactions within the cellular microenvironment.

## 2. Materials and Methods

### 2.1. Production of blood products

Whole blood was collected from healthy blood donors by the National Blood Transfusion Service (Malta) and processed to produce RCCs, plasma, BCs, and platelet concentrates. Unused BC, surplus plasma, and expired platelet concentrates were used for this study.

### 2.2. Filtered plasma (FP) supplement preparation

FP was filtered through a leukocyte filter, aliquoted, and centrifuged (Eppendorf GmbH, Germany) at 3000× *g* for 10 min at 4°C. The centrifuged plasma was then transferred to 50-mL centrifuge tubes and frozen at −20°C. Once needed, the FP was thawed at 37°C and centrifuged at 3000× *g* for 10 min at room temperature (RT). Whenever FP was used, 100 µL of heparin (5,000 IU/mL; Wockhardt, Cat. No. PL 29831) was added per 50 mL of complete culture medium.

### 2.3. Cryoreduced frozen plasma (CRFP) supplement preparation

FFP was thawed overnight at 4°C to remove as much of the cryoprecipitate as possible. The resulting product was termed CRFP. The supernatant was aliquoted into 50-mL centrifuge tubes under aseptic conditions, centrifuged at 3000× *g* for 10 min at 4°C, and stored at −20°C. Since residual cryoprecipitate may still be present, 100 µL of heparin (5,000 IU/mL) was added to 50 mL of complete culture medium when CRFP was used. Alternatively, once the CRFP aliquot was thawed and ready for use, 300 µL of heparin (5000 IU/mL) was pre-added to the plasma aliquot (volume of approximately 40 mL).

### 2.4. Cryodepleted plasma (CDP) supplement preparation

The method of preparing CDP was adapted from Muraglia *et al*. [5], who used lyophilized plasma as a form of culture medium supplement. To prepare CDP, FFP was thawed overnight at 4°C. Under aseptic conditions, the supernatant was collected (approximately 250 mL), supplemented with 5 mL of sterile (filtered) 5.5 M calcium chloride (CaCl_2_; Sigma-Aldrich, Cat. No. C3306) stock solution, aliquoted, and placed in a water bath at 37°C for 3 h. The CaCl_2_ and heat-induced coagulation in turn precipitated residual clotting factors in the FFP. The plasma was then centrifuged at 3000× *g* for 10 min at 4°C and stored at −20°C. Once required, CDP was thawed at 37°C and centrifuged at 3000× *g* for 10 min at 4°C. It is recommended to re-centrifuge the CDP at these settings before every use.

### 2.5. Platelet lysate preparation

Pooled platelets that were no older than 7 days were considered for preparing the lysate. Before lysis, a platelet count using an automated cell counter (Sysmex Europe, Germany) was performed to determine the starting concentration of the selected batch of platelet concentrate. Under aseptic conditions, the platelet concentrate was aliquoted into 50-mL centrifuge tubes and centrifuged at 300× *g* for 10 min at 4°C. The supernatant was discarded, the pellets were resuspended in 20 mL of sterile phosphate-buffered saline (PBS; Sigma-Aldrich, Cat. No. P3813), and centrifuged as before. Washing was repeated twice. Pellets were pooled together before the last wash. Next, 1 mL of PBS per gram of platelets was added to resuspend the combined pellet. Aliquots of 1 mL were prepared and centrifuged to remove the PBS, after which 1 mL of sterile water was added to resuspend the pellet. Pellets were divided into three sets. The first set was processed using the freeze-thaw method, as described by Muraglia *et al*. [5]. The second set was homogenized using a pre-chilled Qiagen TissueLyser LT tissue homogenizer bead mill with 5-mm metallic beads (Qiagen, Cat. No. 69980). The third set was placed in a bath sonicator (Grant Instruments, U.K.) for 30 min. The product of all three methods was then vortexed thoroughly to ensure membrane perturbation and centrifuged at 20,000× *g* for 10 min at 4°C. The supernatant was collected in a new tube, and the protein concentration measured by spectrophotometry using a BioPhotometer (Eppendorf, Germany) and calculated from the absorbance at 280 nm using the Beer–Lambert equation and the extinction coefficient for human serum albumin (35,700 M^−1^ cm^−1^). The lysate was stored at −80°C until used.

### 2.6. TGF beta (TGF-β) quantification analysis

The concentration of TGF-β present in FP, CRFP, CDP, platelet lysate, and fetal bovine serum (FBS) was determined using Human TGF-beta1 DuoSet (Bio-Techne, Cat. No. DY240-05) following the manufacturer’s instructions.

### 2.7. Complete culture medium preparation and cell lines used for testing

The basal medium used consisted of Dulbecco’s Modified Eagle Medium: Nutrient Mixture F-12 (DMEM: F12) with phenol red (Sigma-Aldrich Co, Cat. No. D5523), which was supplemented with 1% (w/v) penicillin/streptomycin (10,000 U/mL penicillin and 10 mg/mL streptomycin in 0.9% NaCl, Sigma-Aldrich, Cat. No. P0781). The concentrations of FP, CRFP, and CDP used for testing were 5%, 10%, or 20%. The concentration of platelet lysate used for testing was 0.1 mg/mL, 0.5 mg/mL, or 1.0 mg/mL. Platelet lysate was added to the basal medium containing CDP immediately before use. Once thawed, lysate aliquots were not re-frozen.

The cultured cell lines (Table 1) on which testing were performed consisted of mesenchymal stem cells (MSCs) either for sub-culturing or for chondrogenic differentiation, SH-SY5Y (human neuroblastoma cell line), and SAOS2 (human osteosarcoma cell line).

**Table 1:** Cells cultured for the various blood component testing.

Fibrin clots	Plasma	Platelet lysate
MSCs – for sub-culturing	MSCs – for sub-culturing	MSCs – for sub-culturing

MSC – for chondrogenic differentiation	SH-SY5Y	SH-SY5Y

SAOS2	SAOS2	SAOS2

### 2.8. MSC collection and culture

Ethics approval for the usage of umbilical cords was granted by the University of Malta Ethics Research Committee (Ref. 90/2016). Cord segments were obtained after full-term delivery from the Obstetrics and Gynaecology ward at Mater Dei Hospital, Malta. MSCs were extracted from Wharton’s jelly as per the protocol, as described by Zammit and Baron [6], transferred into treated 12-well plates, and cultured in complete DMEM: F12 medium using 20% CDP [7].

### 2.9. MSC chondrocyte differentiation

Once the MSCs in the 12-well plates reached an adequate degree of confluence (approximately 40%), the culture medium was substituted by chondrogenic differentiation medium (R&D Systems, Cat. No. SC006) consisting of DMEM: F12, 1% (v/v) penicillin/streptomycin, 1% ITS supplement (containing insulin, transferrin, and selenious acid), and 1% (v/v) chondrogenic supplement. The differentiation medium was changed every 4-5 days. Cells were incubated at 37°C with 5% CO_2_ in a humidified environment. After 21 days, cells were stained with 500 μL of Alcian Blue (100 mg Alcian Blue in 60 mL of ethanol and 40 mL acetic acid; Sigma-Aldrich, Cat. No. A5268). This detected glycosaminoglycans, which comprise part of the extracellular matrix of cartilage. Before staining, all wells were gently washed once with 500 μL of PBS at RT, after which cells were fixed in 500 μL of Tokuda-Baron Fix for 15 min [8]. Following fixation, the wells were washed twice using 500 μL of PBS at RT. Cells were stained for 30 min using 500 μL of Alcian Blue previously diluted in distilled water in a ratio of 1:3. The wells were subsequently washed gently with 500 μL of distilled water. Finally, wells were topped with 500 μL of distilled water and examined by light microscopy for the presence of glycosaminoglycans.

### 2.10. Preparation of fibrin clots

Each batch of FP was first assessed for its coagulation properties. The coagulation tests performed included prothrombin time, international normalized ratio, activated partial thromboplastin time (APTT), APTT ratio, fibrinogen, thrombin time (TT), and factor II activity. The assays were performed by the Coagulation Laboratory of the Pathology Department at Mater Dei Hospital, Malta. DMEM: F12 supplemented with plasma at a ratio ranging from 1:4 to 4:1 was transferred into a 12-well plate, followed by the addition of 25 μL of 0.025 M CaCl_2_ per well. The plate was then incubated at 37°C for 30 min or until the medium solidified (up to overnight), forming a gel. The cells of interest were then resuspended in DMEM: F12 medium not supplemented with plasma or antibiotics, added on top of the gel, and cultured for up to 2 weeks with medium changes every 3-4 days. Dissociation of the fibrin clots at the end of the culturing process was performed using 0.002 M EDTA in PBS alone or in combination with 0.05% trypsin (Sigma Aldrich, Cat. No. 59418C), followed by 5-min incubation at 37°C.

### 2.11. WBC isolation

BC was aseptically transferred to four individual 15-mL centrifuge tubes and centrifuged for 10 min at 1000× *g*. The resultant mononuclear layers were pooled aseptically in a 50-mL centrifuge tube and washed with erythrocyte lysis buffer at a dilution of 1:10 (v/v) during gentle rocking for 10 min. The mixture was subsequently centrifuged at 400× *g* for 10 min at RT, after which the supernatant was discarded. The washing step with erythrocyte lysis buffer was repeated twice more or until a clear pellet was obtained. After the final wash, the pellet was resuspended in 10 mL of PBS at RT and centrifuged at 400× *g* for 10 min at RT. The pellet was resuspended in 1 mL of DMEM, and the WBCs were counted and then seeded at the desired density. Cells were cultured in DMEM:F12 medium supplemented with 55% of any one of the plasma preparations.

### 2.12. WBC culturing and treatment with phytohemagglutinin

The WBCs were seeded at a density of 1 × 10^6^-1 × 10^7^ cells/mL (total volume 1 mL) in a 12-well plate, in triplicate, depending on the experimental set-up being tested. To the cells in the test condition, 2-5 µg/mL of phytohemagglutinin (PHA; Sigma-Aldrich, Cat. No. 11082132001) dissolved in DMEM: F12 medium was added, while to the cells in the control condition an equal volume of basal DMEM: F12 medium were added [9]. WBCs were kept in culture for up to 7 days with the time points for cell harvest being day 0 and day 7. Once harvested, the WBCs were washed once in PBS at RT to remove any remaining medium and serum, and the pellets were stored at −80°C until needed for mRNA extraction.

### 2.13. RNA extraction and cDNA synthesis

Total mRNA was extracted from WBCs using the SV Total RNA Isolation System (Promega, Cat. No. Z3100) following the manufacturer’s instructions. In short, 175 µL of RNA lysis buffer was added to the washed cells and vortexed to ensure complete dissolution of the pellet. The cell lysate was passed 5 times through a 20-gauge needle to shear the genomic DNA and transferred to a 1.5-mL microcentrifuge tube, to which 350 µL of RNA dilution buffer was added and thoroughly mixed by inverting 3-4 times. The tube was placed in a heat block at 70°C for 3 min and then centrifuged at 14,000× *g* for 10 min at RT. The supernatant was transferred to a fresh microcentrifuge tube. Next, 200 µL of 95% ethanol was added and mixed 3-4 times by pipetting. The supernatant was transferred into a spin column, centrifuged at 14,000× *g* for 1 min, washed with 600 µL of RNA wash buffer, centrifuged at 14,000× *g* for 1 min, washed with another 250 µL of RNA wash buffer, and centrifuged at 22,000× *g* for 2 min. The spin column was transferred to a fresh 1.5-mL microcentrifuge tube, and the RNA was eluted in 100 µL of nuclease-free water by centrifugation at 22,000× *g* for 1 min. The RNA concentration was determined spectrophotometrically at 260 nm with an Eppendorf BioPhotometer (Eppendorf, Germany) and stored at −80°C.

The cDNA was synthesized from 5 µg of extracted RNA using the GoScript Reverse Transcriptase System (Promega, Cat No. A5000) following the manufacturer’s instructions. Briefly, the mRNA and primer mixture was prepared by mixing 4 µL of sample RNA and 1 µL of random primer mix. The mixture was vortexed, incubated in a pre-heated heating block at 70°C for 5 min, centrifuged for 10 s at 22,000× *g*, and kept on ice until the reverse transcription mix was prepared. The reverse transcription reaction mix comprised 5.8 µL of nuclease-free water, 4.0 µL of GoScript ×5 reaction buffer, 1.2 µL of MgCl_2_, 1.0 µL of polymerase chain reaction (PCR) nucleotide mix, 2.0 µL of recombinant RNasin Ribonuclease inhibitor, and 1.0 µL of GoScript Reverse Transcriptase. Next, 5 µL of the RNA and primers mix was added to 15 µL of reverse transcription reaction mix. The cDNA synthesis was performed using the following run parameters: Denaturation at 95°C for 5 min, annealing at 55°C for 60 min, and extension at 70°C for 15 min.

### 2.14. Real-time PCR (RT-PCR)

For each PCR reaction, the mix was as follows: 5.0 μL of ×2 QuantiTect SYBR Green PCR Master Mix (Qiagen, Netherlands), 0.5 μL of each primer, and 3.0 μL of RNase-free water. After vortexing, 9 μL of the mixture was dispensed into a PCR tube, to which 1 μL of the template cDNA was added.

The primer pairs used were:

CD4_F: GAACTGACCTCTACAGCTTCC CD4_R: ACCTCC T CCTTCTGGTCCTCC;

CD45_F: GTGTTTCATCAGTACAGACG, CD45_R: GTTG TG GTTGAAATGACAGC;

GAPDH_F: GAAGGTGAAGGTCGGAGTCA, GAPDH_R: GA AGATGGTGATGGGATTTC;

18S_F: CGGCTACCACATCCAAGGAA 18S_R: GCTGGA ATT ACCGCGGCT.

Primers were synthesized by Integrated DNA Technologies (Belgium). RT-PCR was performed using a Rotor-Gene Q (Qiagen, the Netherlands) and the following run parameters: Initial denaturation at 94°C for 5 min, 40 cycles of denaturation at 94°C for 15 s, annealing at 50°C for 30 s, and extension at 72°C for 30 s, followed by a high-resolution melting curve analysis at 1°C/s from 55 to 95°C.

The CD45 marker was selected to monitor the overall leukocyte numbers, while CD4 was chosen as a marker for T-cells. The fold-change in mRNA expression of the CD4 and CD45 genes between PHA-treated leukocytes and untreated controls was used as an indication of T-cell proliferation. The fold-change value was obtained using the relative quantitation 2^−∆∆Ct^ method, with the Cq values of the target genes normalized against glyceraldehyde 3-phosphate dehydrogenase (GAPDH) Cq results as an internal control (IC).

## 3. Results

### 3.1. Plasma supplement preparations

The first stage was to replace the FBS commonly used in cell culture with human plasma. Three different plasma processing methods were used and compared. The concentrations of FP, CRFP, and CDP selected for testing were 5%, 10%, and 20% since these are the ranges most often quoted in the literature, depending on the type of cell and experiment being performed. Although the main focus of this research was on MSCs, there are experiments that require coculturing or the use of the conditioned medium, which is also required to be xeno-free. For this reason, SH-SY5Y and SAOS2 cells were also cultured under the abovementioned conditions.

**Figure 1:**
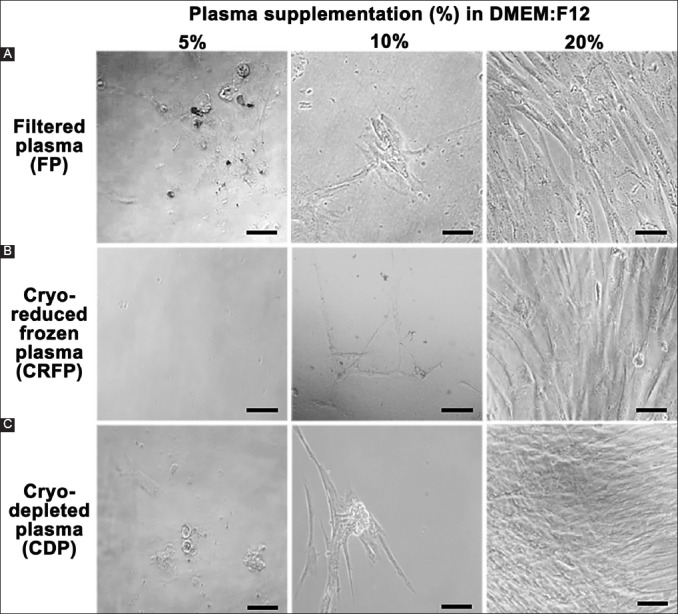
Mesenchymal stem cells cultured in 5, 10, and 20% of different plasma preparations. The scale bar indicates 20 μm.

The cultures of both SH-SY5Y and SAOS2 cells could be easily propagated with all three percentages of plasma supplementation (data not shown). However, MSCs showed great difficulty in proliferating at the lower two concentrations of plasma and only showed a satisfactory level of proliferation at an inclusion level of 20% (Figure 1). This was expected, as MSCs (like all other primary cultures) are much more sensitive to growth conditions than cancer cells. Another point worth mentioning is that all three methods of preparation yielded similar results, implying that the fraction of protein removed as “cryo” on thawing is not particularly rich in growth-supporting factors.

To use the plasma for medium supplementation, it must be kept liquid and, as implied by the name, it was not depleted of its clotting factors. As such, plasma can still result in clotting on exposure to the calcium within the basal media (in this case, DMEM: F12). To prevent this, heparin or CaCl_2_ was added. Since heparin is not always synthetic and xeno-free, the best way to prevent coagulation of the plasma was to add 0.25M CaCl_2_ to precipitate the coagulation factors. A note of caution when using the latter method for culturing cell lines which secrete calcium, as in the case of osteolineage cells such as SAOS2 cells, is that although the calcium added should be enough to remove the majority of the clotting factors, over time the cells might still surround themselves in a gelatinous layer. The addition of 0.002 M EDTA in PBS is suggested to dissolve the overlay on the cells before attempting trypsinization.

### 3.2. Platelet lysate

The second stage was to provide growth factor supplementation in addition to that provided by the plasma since the available plasma from blood establishments will always be from adults. The supplementation provided by the F12 already aided to a large degree the cell proliferation. Considering the push by commercial suppliers to provide special supplements for cell culture, it was decided to include platelet lysate, which was mainly added for its TGF-β content.

Three methods of preparation were tested to determine whether exposure of the platelets to different treatments might have an effect on the type or amount of protein recovered. The highest yield of proteins from platelets was obtained by means of mechanical disruption, which also drastically reduced preparation time.

The concentrations of platelet lysate tested were 0.1 mg/mL, 0.5 mg/mL, and 1.0 mg/mL. These concentrations were selected on the notion that it would be much easier to base the inclusion ratio on the total protein content. This allowed taking into consideration that the lysate contains a complex mixture of different factors at different concentrations, such as TGF-β. Such variation in factor concentration would entail that an ELISA is performed following every single extraction rather than a simple spectrophotometric protein concentration determination. The overall cost and time required would defeat the purpose of the practice shift being proposed.

**Figure 2:**
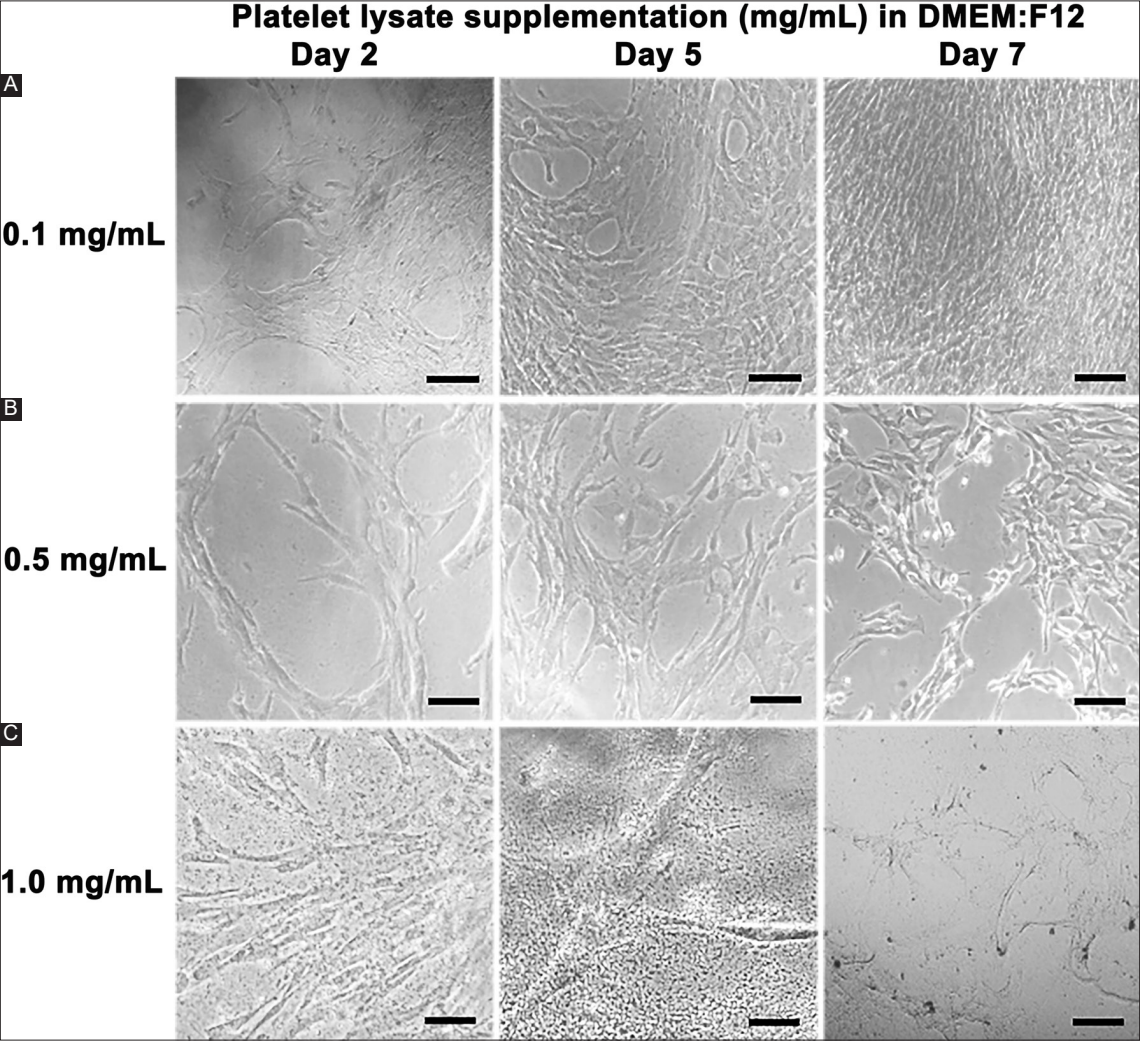
Mesenchymal stem cells cultured in 0.1-1.0 mg/mL platelet lysate over 7 days. The scale bar indicates 20 μm.

It was evident for all cell types tested and especially for MSCs (Figure 2) that, although such supplementation is beneficial at low concentrations (0.1 mg/mL), the cells died at higher levels of a platelet lysate, usually within 2-4 days.

### 3.3. TGF-β quantification analysis

Since one of the major reasons for preparing the platelet lysate supplement was to boost TGF-β^-^ signaling, the concentration of TGF-β within the various supplementations was determined by ELISA (Table 2).

**Table 2:** TGF-β concentration of the various blood product preparations.

Product	Concentration [pg/mL]
Filtered plasma	167.39-196.38

Cryoreduced frozen plasma	163.80-201.45

Cryodepleted plasma	147.83-172.46

Commercial FBS	277.54-423.91

Freeze-thawed platelet lysate	1,147.32-1,693.48

Sonicated platelet lysate	1,040.22-1,255.07

Homogenized platelet lysate	1,000.00-1,390.58

TGF-β: Transforming growth factor, FBS: Fetal bovine serum

As clearly observable from the tabulated values, the target average concentration is around 300 pg/mL, which is what is generally achieved when using a 10% inclusion of commercial FBS. All the plasma preparations presented values that are half the values obtained for commercial FBS. It therefore stands to reason that a 20% inclusion ratio would be required by the cells to proliferate at a reasonable rate. The platelet lysate preparations, irrespective of the method implemented, contained considerably high TGF-β concentrations per mL, making them ideal choices for such a purpose.

### 3.4. Fibrin clots

The third stage was to attempt to use the plasma as a source for a culture supporting matrix. Considering the extensive studies undertaken to explore either natural or synthetic extracellular matrices, both in terms of physical properties and chemical composition, it was essential to determine the optimal clot density, setting time, and the means by which to dissolve the clot to retrieve the cells within.

Before starting the clot testing, biochemical quantifications were performed on the plasma to determine the clotting properties of the sera available (Table 3). All the values obtained fall within the expected range insofar as blood donors are healthy people and therefore should not present any coagulopathies. Thus, the conditions that were determined hereunder may be universally applied to any plasma pool that is intended to be used in the production of fibrin clots.

**Table 3:** Ranges for coagulation properties of human sera used to produce the fibrin clots.

Outcome parameter	Range
PT (s)	10.3-13.0

INR ratio	0.99-1.25

APTT (s)	28.5-34.5

APTT ratio	0.96-1.16

Fibrinogen (g/L)	2.27-3.26

Thrombin time (s)	12.3-14.0

FII activity (%)	78.3-119.7

PT: Prothrombin time, INR: International normalized ratio, APTT: Activated partial thromboplastin time, TT: Thrombin time, FII: Factor II

It was decided that the easiest setting condition would be achieved by the addition of 0.025 M CaCl_2_. To determine the range of usable matrix densities, the medium to plasma ratios tested were 4:1, 3:1, 2:1, 1:1, 1:2, 1:3, and 1:4. The higher the proportion of plasma within the mix, the higher the rigidity of the clot formed, with the ideal ratio being that of 3:1. For rigid clots (high plasma content), more calcium was required for all the plasma to solidify or else it would just form a semi-solid, which could not support the cells. However, this also led to longer setting time. The setting time for each clot was determined in minutes, following the addition of 0.025 M CaCl_2_. The average setting time was 20 min, although the higher plasma ratios (1:2, 1:3, and 1:4) required an overnight incubation at 37°C with an additional 25 μL of CaCl_2_ to gel over evenly. In the case of clots that coagulated in a short time frame, cells could be mixed with the medium and suspended evenly. However, in the case of clots whose coagulation speed was lower than the sedimentation rate of the seeded cells, these were better suited as a coating of the wells, with cells growing sandwiched between two clots. Before seeding, cells were suspended in a similar fibrin clot ratio and only then added to the plate containing the previously formed clot.

**Figure 3:**
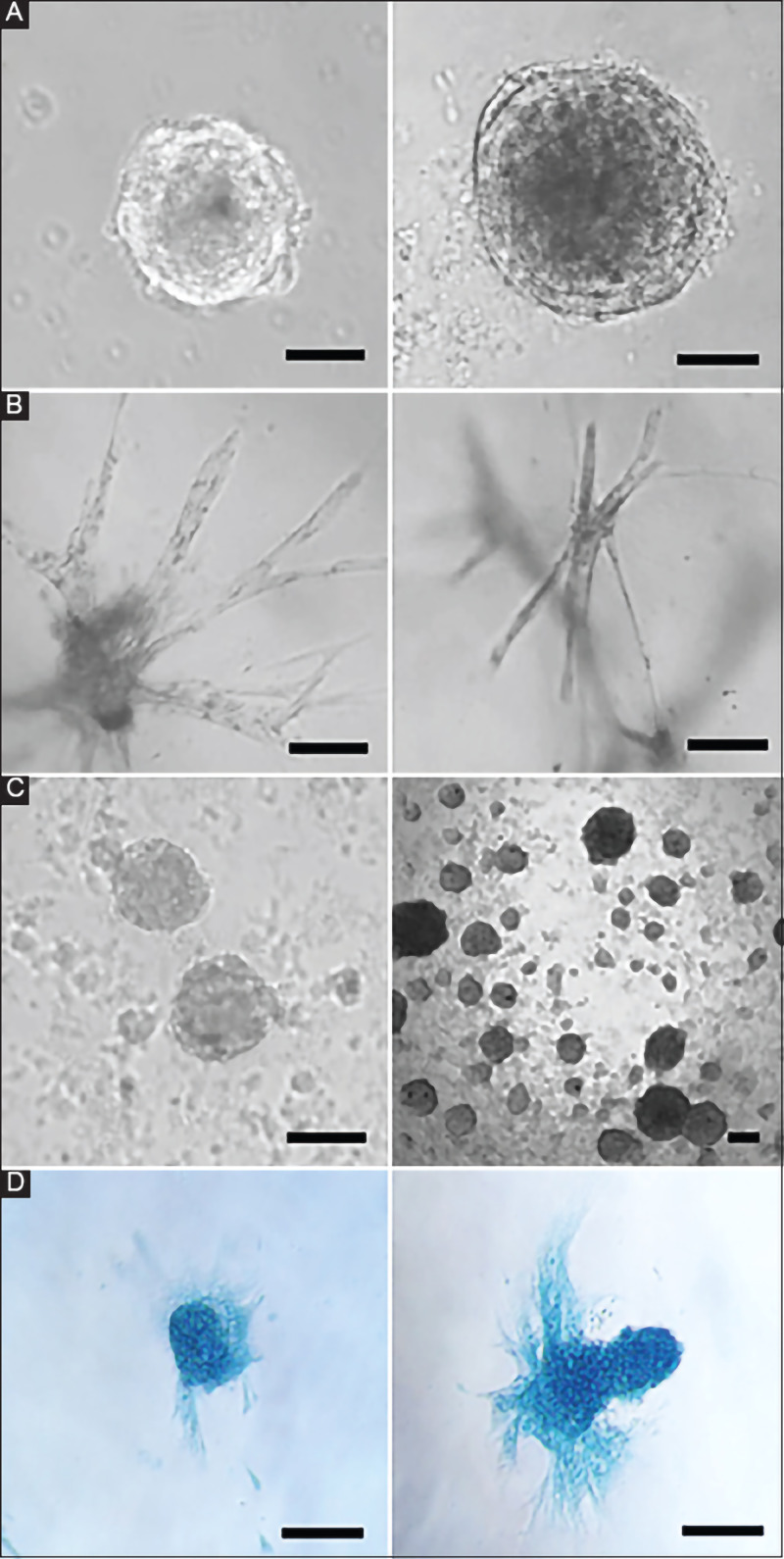
(**A**) MSCs in low-density clots, (**B**) MSCs in high-density clots, (**C**) cancer spheroids in low-density clots, (**D**) MSCs in chondrocyte differentiation medium. The scale bar indicates 20 μm.

MSCs and cancer cells were followed over a culturing period of up to 2 weeks. The effect of clot density on cell proliferation and cellular structure was clearly evident, with cells in lower density clots forming spheroids while those in higher density clots formed spindles along the lines of least stress (Figure 3). The overall size of the spheroids was also affected by clot density, with smaller spheroids being formed at higher clot densities. One issue encountered with low-density clots was that the increased weight of the spheroids caused them to sink through the clot and, upon touching the bottom of the well, spread out in 2D.

At the end of culturing, it was essential to establish a reliable way by which to dissolving the clot and retrieving the cells without damaging them in the process. The use of 0.05% trypsin directly on the clots had very little effect, even with extended incubation times due to the high amount of protein present. The dissolution of the clots was much easier using 0.002 M EDTA, which then allowed a subsequent treatment with trypsin to partly break down the spheroids and allow subculturing.

### 3.5. RT-PCR analysis of leukocyte cultures

BCs and leukocytes available as a transfusion by-product can be used for a wide variety of experiments. However, the main concern was the extent of their viability through time and their ability to act like leukocytes *in vivo*. For this reason, leukocytes were seeded at a density of 1 × 10^6^-1 × 10^7^ cells/mL (which was as close as possible in static 2D culture to the concentration in blood) and treated with PHA, a known lymphocyte stimulant, at a concentration of 2-5 µg/mL. An experimental period of 7 days was selected because it was the longest available time before a sizable decrease in cell viability was recorded.

RT-PCR was performed in technical triplicates on two batches of leukocytes (biological replicates), both extracted from BCs. There was a noticeable discrepancy in the mRNA expression change for CD4 between experiments, while reproducible results were obtained for CD45 (Table 4). This means that the total number of CD4^+^ cells over 7 days changes reproducibly, however the number of proliferating T-cells does not and depends on the initial number of T-cells and the number of cells stimulated by the PHA treatment.

**Table 4:** CD4 and CD45 mRNA expression fold-change between PHA-treated leukocytes and untreated control.

mRNA	Experiment	Fold-change
CD4	Exp. 1	2.92 (1.88-4.95)

	Exp. 2	−1.18 (−1.45-1.05)

CD45	Exp. 1	2.36 (1.82-3.44)

	Exp. 2	3.64 (2.79-4.75)

Since GAPDH is not always considered a reliable IC, the 2^−∆∆Ct^ of the second run (Exp. 2) was also calculated using 18S as an IC. The 18S ribosomal component was chosen because its expression should be fairly stable being a basic component of any eukaryotic cell. The results obtained using 18S as IC gave comparable fold-change values (Table 5).

**Table 5:** CD4 and CD45 mRNA expression fold-change between PHA-treated leukocytes and untreated control (for experiment 2 results using 18S as IC).

mRNA	Fold-change
CD4	−1.23 (−1.54-1.02)

CD45	3.50 (2.65-4.64)

Despite the match in IC fold changes, a final consideration was given to the fold-change in GAPDH mRNA expression between the PHA-treated leukocytes and untreated control as an indication of the cellular condition at the end of the experimental run. A significant difference was determined between the two runs (Table 6). The difference in GAPDH expression between experiments seems to indicate that conditions other than the experimental treatment are having an impact on the leukocytes over the 7-day period, and thus need to be monitored and factored into the calculations. The similarity in mRNA expression fold-change observed between GAPDH and 18S indicates the possibility that one major factor is at play, affecting cells in a fundamental way, so as to alter both GAPDH and 18S expression to a similar extent.

**Table 6:** GAPDH mRNA expression fold-change comparison between PHA-treated leukocytes and untreated control.

Experiment No.	mRNA	Fold-change
Exp. 1	GAPDH	−4.88 (−7.03-−3.39)

Exp. 2	GAPDH	5.31 (5.22-5.41)

## 4. Discussion

**Figure 4:**
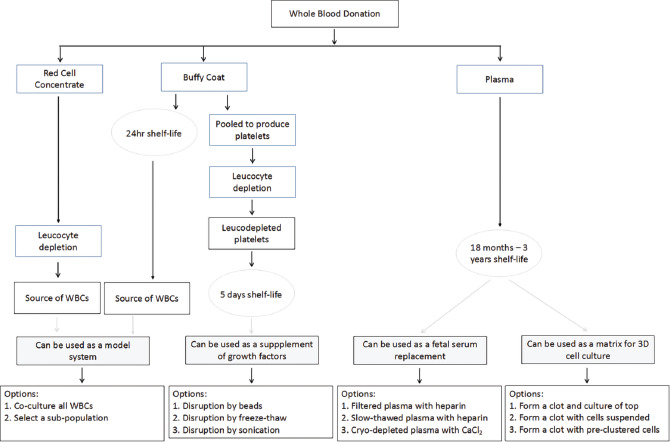
Summary of the process flow by which blood products are prepared for transfusion and then redirected for research.

The scope of this research was to take the blood components, i.e., plasma and BCs (including platelets), which has not been transfused (and thus wasted) and redirect the use of these products toward their potential application in xeno-free research. We hereby show a number of possible routes by which this can be done and a number of applications for these products (Figure 4). The application for stem cell culturing is of particular interest for studying cellular behavior in a way that mimics the *in vivo* condition as closely as possible, as exemplified by 3-D cell and organoid cultures.

The first blood product redirected for research was plasma, which was intended to be used as a supplement to replace FBS. DMEM: F12 was selected as the basal medium for these experiments due to the additional supplementation provided by the F-12. The DMEM: F12 formulation tends to be the preferred basal medium of stem cell culture by numerous research groups and commercial suppliers [3]. The data indicated that FP, CRFP, and CDP are equally suitable for replacing FBS, with the percentage supplementation required depending exclusively on the type of cell being cultured. MSCs isolated from placental cords were the main focus of the research and yielded a fair number of cells at 20% supplementation with human plasma, without changes in morphology or stemness potential. The main growth factor of interest, TGF-β, was quantified, and its levels in donor sera were roughly half of those in commercial FBS. Once clotting factors had been dealt with, the removal of the cryoprecipitate through the addition of CaCl_2_ resulted in debris-free medium, without having any adverse effect on cell proliferation. Overall, these experiments showed that human-derived plasma preparations are a viable option to the commercially available, poorly defined, animal-derived sera. Further work is planned to quantify other growth factors within human donor plasma and compared these to the growth factor profiles of FBS.

The next blood product processed was the platelet concentrate. Platelet lysate was produced, which was intended to be used as a growth factor supplement. The TGF-β assay showed over 5 times higher content in platelet lysate than in FBS, making it an excellent supplement for plasma at low doses. The most optimal dose tested was 0.1 mg/mL, at which any detrimental effects on the cultured cells were avoided. The highest yield of proteins in platelet lysate was obtained when using mechanical disruption, which also had the shortest preparation time. Although this technique might not be available to all laboratories, the yields obtained from all three methods are not significantly different and therefore the “lower” efficiency of the other two methods in retrieving protein in the lysate would not be an issue and would not result in limited supplementation for any given initial amount of platelets. Once again, further work will be performed to quantify other growth factors in the platelet lysate and compare these to FBS.

Subsequently, fibrin clots were generated from human plasma to act as 3D cell culture systems, making them completely xeno-free, and a much closer physiological model. The optimal medium-to-plasma ratio was found to be 3:1, with such clots having an approximate setting time of 20-30 min. High plasma ratios were found to clot poorly, and although this improved when an extra 25 µL of CaCl_2_ was added and incubation was performed overnight, the clots formed were not even. To avoid cells settling on the bottom and expanding in 2D, a first gel layer (enough to fill a fourth of the well depth) should be set. Subsequently, the cells mixed with plasma, DMEM, and CaCl_2_ should be added as a second gel layer. This ensures cells will be trapped in the second layer, or at least provide enough time for the experiment to be performed while the cells are traversing the gel layers. This method is still not fail-proof when using a large seeding density, large cells, or cancer cells with high migration potential. The clots were dissolved most easily by treating with 0.002 M EDTA.

The final blood product prepared was the leukocyte culture, in which multiple leukocyte sub-types can be studied in coculture, in a xeno-free system, using human plasma (55% being the average plasma concentration found naturally in peripheral blood) and platelet lysate supplementation. This model thus provides a cost-effective, simple platform to conduct culture-dependent experiments while allowing the different cell subtypes to interact, mimicking the natural blood micro-environment. The culture conditions can be amended accordingly, for example, by adding cofactors that induce selective stimulation of a particular cell sub-type, depending on the experiment needs. In the experiments performed here, RT-PCR was used to measure the effect of PHA, a known immune cell stimulant, on CD4^+^ T-cells.

The results obtained in Experiment 1 indicated that this model produced the expected results (i.e., increase in CD4 mRNA expression together with an overall increase in CD45 mRNA expression in the presence of PHA). However, Experiment 2 did not produce the expected change in CD4 and analysis of the ICs revealed that the mRNA expression of the IC itself differed greatly between experimental runs, possibly causing the difference in CD4 result analysis. The differences in IC expression between experiments, and the difference in expression between the IC in the PHA-treated cells and untreated control cells, require further validation of the ICs used in this model.

GAPDH and 18S are both widely used ICs in various RT-PCR models and were even suggested as ICs for use specifically with leukocytes [10]. The IC expression results obtained in this study suggest that possibly both GAPDH and 18S are affected by external factors during the experiment, such as differences in cell life-cycle stages [11] or the changing interaction between different leukocyte sub-types over the culturing period. Other genes can be tested as ICs in an attempt to find the right candidates that show minimal expression change in a variety of conditions. The use of an exogenous *in vitro* transcribed control is not suggested since the RNA from an exogenous source is not copurified and, more importantly, it does not reflect the transcriptional activity within cells [12]. Another approach is to use absolute quantification RT-PCR. However, this requires a larger investment both in resources and validation time.

## 5. Conclusions

Blood transfusion wastes and by-products were used to culture MSCs in a xeno-free system. With the basal medium DMEM: F12, the highest proliferation rate was obtained using 20% human plasma and 0.1 mg/mL platelet lysate supplementation. On the other hand, tumor cell lines could be cultured with just 5-10% human plasma. Moreover, the preparation of CDP generated less debris, allowing cells to grow in a better environment. Both plasma and lysate provided an adequate concentration of TGF-β to the cells in culture. The best fibrin clot density was achieved at a ratio of 3-parts medium to 1-part plasma, with a clotting time of 20-30 min. Clots were then dissolved using 0.002 M EDTA.

The leukocyte mixed-culture model shows the potential for use in various experimental settings. A realistic culture model mimicking the natural blood micro-environment can be obtained using the aforementioned blood products without investment in expensive reagents or equipment. Notwithstanding the variation and quantity of different cells in culture, the model is able to detect the changes induced by the experimental condition. Further validation of the analysis method described here is needed to ensure proper interpretation of results obtained.

The use of blood establishment wastes and by-products reduces the reliance on commercially available, ready-made products for xeno-free cell culture, the contents of which are not described to the customers. Although there are undoubtedly benefits in terms of both cost and applications, the use of these products still necessitates further in-house characterization and optimization for the reproducibility of results. The cost of such specialist media greatly limits the output of stem cell research groups with limited budgets that could easily benefit from the proposed applications by collaborating with a blood establishment at very little additional cost to either party. In so doing, the opportunities and potential for therapeutic stem cell research are greatly enhanced.
